# Supplementary material for: Resolving the full spectrum of human genome variation using Linked-Reads
Source: Genome Res. 2019 Apr;29(4):635–45. doi: 10.1101/gr.234443.118 (PMC6442396; doi:10.1101/gr.234443.118)
Supplement: Supplemental Material [file supp_gr.234443.118_Supplemental_longranger-2.2.zip › longranger-2.2.tar.bz2!/lr2.2-public/lib/go/src/code.google.com/vitess/docs/getting-started/local-instance.html]

Vitess / Running Vitess on a Local Server


Toggle navigation

- Overview
- Guides
- Reference
- Blog
- GitHub

- #### Overview

  - What is Vitess
  - Scaling MySQL with Vitess
  - Key Concepts
- #### Getting Started

  - Run Vitess on Kubernetes
    - Custom Docker Build
  - Run Vitess Locally
- #### User Guide

  - Introduction- Client Libraries- Backing Up Data- Reparenting
        - Schema Management
        - VSchema Guide
        - Schema Swap (Tutorial)
        - Sharding
          - Horizontal Sharding (Tutorial, automated)
          - Horizontal Sharding (Tutorial, manual)
          - Sharding in Kubernetes (Tutorial, automated)
          - Sharding in Kubernetes (Tutorial, manual)
        - Vitess Sequences
        - MySQL Server Protocol
        - Vitess and Replication
        - Update Stream
        - Row Based Replication
        - Topology Service
        - Transport Security Model
        - Launching
          - Scalability Philosophy
          - Production Planning
          - Server Configuration
          - 2PC Guide
          - Troubleshooting
        - Upgrading
- #### Reference Guides

  - Vitess API- vtctl Commands
- #### Other Resources

  - Presentations- Blog- Roadmap
- #### Contributing

  - Contributing to Vitess- GitHub Workflow- Code Reviews
- GitHub

# Running Vitess on a Local Server

- #### Overview

  - What is Vitess
  - Scaling MySQL with Vitess
  - Key Concepts
- #### Getting Started

  - Run Vitess on Kubernetes
    - Custom Docker Build
  - Run Vitess Locally
- #### User Guide

  - Introduction- Client Libraries- Backing Up Data- Reparenting
        - Schema Management
        - VSchema Guide
        - Schema Swap (Tutorial)
        - Sharding
          - Horizontal Sharding (Tutorial, automated)
          - Horizontal Sharding (Tutorial, manual)
          - Sharding in Kubernetes (Tutorial, automated)
          - Sharding in Kubernetes (Tutorial, manual)
        - Vitess Sequences
        - MySQL Server Protocol
        - Vitess and Replication
        - Update Stream
        - Row Based Replication
        - Topology Service
        - Transport Security Model
        - Launching
          - Scalability Philosophy
          - Production Planning
          - Server Configuration
          - 2PC Guide
          - Troubleshooting
        - Upgrading
- #### Reference Guides

  - Vitess API- vtctl Commands
- #### Other Resources

  - Presentations- Blog- Roadmap
- #### Contributing

  - Contributing to Vitess- GitHub Workflow- Code Reviews

You can build Vitess using either Docker or a
manual build process.

If you run into issues or have questions, please post on our
forum.

## Docker Build

To run Vitess in Docker, you can either use our pre-built images on Docker Hub, or build them yourself.

### Docker Hub Images

- The vitess/base image contains a full
  development environment, capable of building Vitess and running integration tests.
- The vitess/lite image contains only
  the compiled Vitess binaries, excluding ZooKeeper. It can run Vitess, but
  lacks the environment needed to build Vitess or run tests. It's primarily used
  for the Vitess on Kubernetes guide.

For example, you can directly run `vitess/base`, and Docker will download the
image for you:

```
$ sudo docker run -ti vitess/base bash
vitess@32f187ef9351:/vt/src/github.com/youtube/vitess$ make build
```

Now you can proceed to start a Vitess cluster inside
the Docker container you just started. Note that if you want to access the
servers from outside the container, you'll need to expose the ports as described
in the Docker Engine Reference Guide.

For local testing, you can also access the servers on the local IP address
created for the container by Docker:

```
$ docker inspect 32f187ef9351 | grep IPAddress
### example output:
#    "IPAddress": "172.17.3.1",
```

### Custom Docker Image

You can also build Vitess Docker images yourself to include your
own patches or configuration data. The
Dockerfile
in the root of the Vitess tree builds the `vitess/base` image.
The docker
subdirectory contains scripts for building other images, such as `vitess/lite`.

Our `Makefile` also contains rules to build the images. For example:

```
# Create vitess/bootstrap, which prepares everything up to ./bootstrap.sh
vitess$ make docker_bootstrap
# Create vitess/base from vitess/bootstrap by copying in your local working directory.
vitess$ make docker_base
```

## Manual Build

The following sections explain the process for manually building
Vitess without Docker.

### Install Dependencies

We currently test Vitess regularly on Ubuntu 14.04 (Trusty) and Debian 8 (Jessie).
OS X 10.11 (El Capitan) should work as well, the installation instructions are below.

#### Ubuntu and Debian

In addition, Vitess requires the software and libraries listed below.

1. Install Go 1.8+.
2. Install MariaDB 10.0 or
   MySQL 5.6. You can use any
   installation method (src/bin/rpm/deb), but be sure to include the client
   development headers (`libmariadbclient-dev` or `libmysqlclient-dev`).

   The Vitess development team currently tests against MariaDB 10.0.21
   and MySQL 5.6.27.

   If you are installing MariaDB, note that you must install version 10.0 or
   higher. If you are using `apt-get`, confirm that your repository
   offers an option to install that version. You can also download the source
   directly from mariadb.org.

   If you are using Ubuntu 14.04 with MySQL 5.6, the default install may be
   missing a file too, `/usr/share/mysql/my-default.cnf`. It would show as an
   error like `Could not find my-default.cnf`. If you run into this, just add
   it with the following contents:

   ```
   [mysqld]
   sql_mode=NO_ENGINE_SUBSTITUTION,STRICT_TRANS_TABLES
   ```
3. Select a lock service from the options listed below. It is technically
   possible to use another lock server, but plugins currently exist only
   for ZooKeeper and etcd.

   - ZooKeeper 3.3.5 is included by default.
   - Install etcd v3.0+.
     If you use etcd, remember to include the `etcd` command
     on your path.
4. Install the following other tools needed to build and run Vitess:

   - make
   - automake
   - libtool
   - python-dev
   - python-virtualenv
   - python-mysqldb
   - libssl-dev
   - g++
   - mercurial
   - git
   - pkg-config
   - bison
   - curl
   - unzip

   These can be installed with the following apt-get command:

   ```
   $ sudo apt-get install make automake libtool python-dev python-virtualenv python-mysqldb libssl-dev g++ mercurial git pkg-config bison curl unzip
   ```
5. If you decided to use ZooKeeper in step 3, you also need to install a
   Java Runtime, such as OpenJDK.

   ```
   $ sudo apt-get install openjdk-7-jre
   ```

#### OS X

1. Install Homebrew. If your /usr/local directory is not empty and you never used Homebrew before,
   it will be
   mandatory
   to run the following command:

   ```
   sudo chown -R $(whoami):admin /usr/local
   ```
2. On OS X, MySQL 5.6 has to be used, MariaDB doesn't work for some reason yet. It should be installed from Homebrew
   (install steps are below).
3. If Xcode is installed (with Console tools, which should be bundled automatically since the 7.1 version), all
   the dev dependencies should be satisfied in this step. If no Xcode is present, it is necessery to install pkg-config.

   ```
   brew install pkg-config
   ```
4. ZooKeeper is used as lock service.
5. Run the following commands:

   ```
   brew install go automake libtool python mercurial git bison curl wget homebrew/versions/mysql56
   pip install --upgrade pip setuptools
   pip install virtualenv
   pip install MySQL-python
   pip install tox
   ```
6. Install Java runtime from this URL: https://support.apple.com/kb/dl1572?locale=en\_US
   Apple only supports Java 6. If you need to install a newer version, this link might be helpful:
   http://osxdaily.com/2015/10/17/how-to-install-java-in-os-x-el-capitan/
7. The Vitess bootstrap script makes some checks for the go runtime, so it is recommended to have the following
   commands in your ~/.profile or ~/.bashrc or ~/.zshrc:

   ```
   export PATH=/usr/local/opt/go/libexec/bin:$PATH
   export GOROOT=/usr/local/opt/go/libexec
   ```
8. There is a problem with installing the enum34 Python package using pip, so the following file has to be edited:
    `/usr/local/opt/python/Frameworks/Python.framework/Versions/2.7/lib/python2.7/distutils/distutils.cfg`

   and this line:

   ```
   prefix=/usr/local
   ```

   has to be commented out:

   ```
   # prefix=/usr/local
   ```

   After running the ./bootstrap.sh script from the next step, you can revert the change.
9. For the Vitess hostname resolving functions to work correctly, a new entry has to be added into the /etc/hosts file
   with the current LAN IP address of the computer (preferably IPv4) and the current hostname, which you get by
   typing the 'hostname' command in the terminal.

   It is also a good idea to put the following line to force the Go DNS resolver
   in your ~/.profile or ~/.bashrc or ~/.zshrc:

   ```
   export GODEBUG=netdns=go
   ```

### Build Vitess

1. Navigate to the directory where you want to download the Vitess
   source code and clone the Vitess Github repo. After doing so,
   navigate to the `src/github.com/youtube/vitess` directory.

   ```
   cd $WORKSPACE
   git clone https://github.com/youtube/vitess.git \
       src/github.com/youtube/vitess
   cd src/github.com/youtube/vitess
   ```
2. Set the `MYSQL_FLAVOR` environment variable. Choose the appropriate
   value for your database. This value is case-sensitive.

   ```
   export MYSQL_FLAVOR=MariaDB
   # or (mandatory for OS X)
   # export MYSQL_FLAVOR=MySQL56
   ```
3. If your selected database installed in a location other than `/usr/bin`,
   set the `VT_MYSQL_ROOT` variable to the root directory of your
   MariaDB installation. For example, if MariaDB is installed in
   `/usr/local/mysql`, run the following command.

   ```
   export VT_MYSQL_ROOT=/usr/local/mysql

   # on OS X, this is the correct value:
   # export VT_MYSQL_ROOT=/usr/local/opt/mysql56
   ```

   Note that the command indicates that the `mysql` executable should
   be found at `/usr/local/mysql/bin/mysql`.
4. Run `mysql_config --version` and confirm that you
   are running the correct version of MariaDB or MySQL. The value should
   be 10 or higher for MariaDB and 5.6.x for MySQL.
5. Build Vitess using the commands below. Note that the
   `bootstrap.sh` script needs to download some dependencies.
   If your machine requires a proxy to access the Internet, you will need
   to set the usual environment variables (e.g. `http_proxy`,
   `https_proxy`, `no_proxy`).

   Run the boostrap.sh script:

   ```
   ./bootstrap.sh
   ### example output:
   # skipping zookeeper build
   # go install golang.org/x/tools/cmd/cover ...
   # Found MariaDB installation in ...
   # creating git pre-commit hooks
   #
   # source dev.env in your shell before building
   ```

   ```
   # Remaining commands to build Vitess
   . ./dev.env
   make build
   ```

### Run Tests

**Note:** If you are using etcd, set the following environment variable:

```
export VT_TEST_FLAGS='--topo-server-flavor=etcd'
```

The default targets when running `make` or `make test` contain a full set of
tests intended to help Vitess developers to verify code changes. Those tests
simulate a small Vitess cluster by launching many servers on the local
machine. To do so, they require a lot of resources; a minimum of 8GB RAM
and SSD is recommended to run the tests.

If you want only to check that Vitess is working in your environment,
you can run a lighter set of tests:

```
make site_test
```

#### Common Test Issues

Attempts to run the full developer test suite (`make` or `make test`)
on an underpowered machine often results in failure. If you still see
the same failures when running the lighter set of tests (`make site_test`),
please let the development team know in the
vitess@googlegroups.com
discussion forum.

##### Node already exists, port in use, etc.

A failed test can leave orphaned processes. If you use the default
settings, you can use the following commands to identify and kill
those processes:

```
pgrep -f -l '(vtdataroot|VTDATAROOT)' # list Vitess processes
pkill -f '(vtdataroot|VTDATAROOT)' # kill Vitess processes
```

##### Too many connections to MySQL, or other timeouts

This error often means your disk is too slow. If you don't have access
to an SSD, you can try testing against a
ramdisk.

##### Connection refused to tablet, MySQL socket not found, etc.

These errors might indicate that the machine ran out of RAM and a server
crashed when trying to allocate more RAM. Some of the heavier tests
require up to 8GB RAM.

##### Connection refused in zkctl test

This error might indicate that the machine does not have a Java Runtime
installed, which is a requirement if you are using ZooKeeper as the lock server.

##### Running out of disk space

Some of the larger tests use up to 4GB of temporary space on disk.

## Start a Vitess cluster

After completing the instructions above to build Vitess,
you can use the example scripts in the Github repo to bring up a Vitess
cluster on your local machine. These scripts use ZooKeeper as the
lock service. ZooKeeper is included in the Vitess distribution.

1. **Check system settings**

   Some Linux distributions ship with default file descriptor limits
   that are too low for database servers. This issue could show up
   as the database crashing with the message "too many open files".

   Check the system-wide `file-max` setting as well as user-specific
   `ulimit` values. We recommend setting them above 100K to be safe.
   The exact procedure
   may vary depending on your Linux distribution.
2. **Configure environment variables**

   If you are still in the same terminal window that
   you used to run the build commands, you can skip to the next
   step since the environment variables will already be set.

   If you're adapting this example to your own deployment, the only environment
   variables required before running the scripts are `VTROOT` and `VTDATAROOT`.

   Set `VTROOT` to the parent of the Vitess source tree. For example, if you
   ran `make build` while in `$HOME/vt/src/github.com/youtube/vitess`,
   then you should set:

   ```
   export VTROOT=$HOME/vt
   ```

   Set `VTDATAROOT` to the directory where you want data files and logs to
   be stored. For example:

   ```
   export VTDATAROOT=$HOME/vtdataroot
   ```
3. **Start ZooKeeper**

   Servers in a Vitess cluster find each other by looking for
   dynamic configuration data stored in a distributed lock
   service. The following script creates a small ZooKeeper cluster:

   ```
   $ cd $VTROOT/src/github.com/youtube/vitess/examples/local
   vitess/examples/local$ ./zk-up.sh
   ### example output:
   # Starting zk servers...
   # Waiting for zk servers to be ready...
   ```

   After the ZooKeeper cluster is running, we only need to tell each
   Vitess process how to connect to ZooKeeper. Then, each process can
   find all of the other Vitess processes by coordinating via ZooKeeper.

   Each of our scripts automatically uses the `TOPOLOGY_FLAGS` environment
   variable to point to the global ZooKeeper instance. The global instance in
   turn is configured to point to the local instance. In our sample scripts,
   they are both hosted in the same ZooKeeper service.
4. **Start vtctld**

   The *vtctld* server provides a web interface that
   displays all of the coordination information stored in ZooKeeper.

   ```
   vitess/examples/local$ ./vtctld-up.sh
   # Starting vtctld
   # Access vtctld web UI at http://localhost:15000
   # Send commands with: vtctlclient -server localhost:15999 ...
   ```

   Open `http://localhost:15000` to verify that
   *vtctld* is running. There won't be any information
   there yet, but the menu should come up, which indicates that
   *vtctld* is running.

   The *vtctld* server also accepts commands from the `vtctlclient` tool,
   which is used to administer the cluster. Note that the port for RPCs
   (in this case `15999`) is different from the web UI port (`15000`).
   These ports can be configured with command-line flags, as demonstrated
   in `vtctld-up.sh`.

   For convenience, we'll use the `lvtctl.sh` script in example commands,
   to avoid having to type the *vtctld* address every time.

   ```
   # List available commands
   vitess/examples/local$ ./lvtctl.sh help
   ```
5. **Start vttablets**

   The `vttablet-up.sh` script brings up three vttablets, and assigns them to
   a keyspace and shard according to the variables
   set at the top of the script file.

   ```
   vitess/examples/local$ ./vttablet-up.sh
   # Output from vttablet-up.sh is below
   # Starting MySQL for tablet test-0000000100...
   # Starting vttablet for test-0000000100...
   # Access tablet test-0000000100 at http://localhost:15100/debug/status
   # Starting MySQL for tablet test-0000000101...
   # Starting vttablet for test-0000000101...
   # Access tablet test-0000000101 at http://localhost:15101/debug/status
   # Starting MySQL for tablet test-0000000102...
   # Starting vttablet for test-0000000102...
   # Access tablet test-0000000102 at http://localhost:15102/debug/status
   ```

   After this command completes, refresh the *vtctld* web UI, and you should
   see a keyspace named `test_keyspace` with a single shard named `0`.
   This is what an unsharded keyspace looks like.

   If you click on the shard box, you'll see a list of tablets in that shard.
   Note that it's normal for the tablets to be unhealthy at this point, since
   you haven't initialized them yet.

   You can also click the **STATUS** link on each tablet to be taken to its
   status page, showing more details on its operation. Every Vitess server has
   a status page served at `/debug/status` on its web port.
6. **Initialize MySQL databases**

   Next, designate one of the tablets to be the initial master.
   Vitess will automatically connect the other slaves' mysqld instances so
   that they start replicating from the master's mysqld.
   This is also when the default database is created. Since our keyspace is
   named `test_keyspace`, the MySQL database will be named `vt_test_keyspace`.

   ```
   vitess/examples/local$ ./lvtctl.sh InitShardMaster -force test_keyspace/0 test-100
   ### example output:
   # master-elect tablet test-0000000100 is not the shard master, proceeding anyway as -force was used
   # master-elect tablet test-0000000100 is not a master in the shard, proceeding anyway as -force was used
   ```

   **Note:** Since this is the first time the shard has been started,
   the tablets are not already doing any replication, and there is no
   existing master. The `InitShardMaster` command above uses the `-force` flag
   to bypass the usual sanity checks that would apply if this wasn't a
   brand new shard.

   After running this command, go back to the **Shard Status** page
   in the *vtctld* web interface. When you refresh the
   page, you should see that one *vttablet* is the master
   and the other two are replicas.

   You can also see this on the command line:

   ```
   vitess/examples/local$ ./lvtctl.sh ListAllTablets test
   ### example output:
   # test-0000000100 test_keyspace 0 master localhost:15100 localhost:33100 []
   # test-0000000101 test_keyspace 0 replica localhost:15101 localhost:33101 []
   # test-0000000102 test_keyspace 0 replica localhost:15102 localhost:33102 []
   ```
7. **Create a table**

   The `vtctlclient` tool can be used to apply the database schema across all
   tablets in a keyspace. The following command creates the table defined in
   the `create_test_table.sql` file:

   ```
   # Make sure to run this from the examples/local dir, so it finds the file.
   vitess/examples/local$ ./lvtctl.sh ApplySchema -sql "$(cat create_test_table.sql)" test_keyspace
   ```

   The SQL to create the table is shown below:

   ```
   CREATE TABLE messages (
     page BIGINT(20) UNSIGNED,
     time_created_ns BIGINT(20) UNSIGNED,
     message VARCHAR(10000),
     PRIMARY KEY (page, time_created_ns)
   ) ENGINE=InnoDB
   ```
8. **Take a backup**

   Now that the initial schema is applied, it's a good time to take the first
   backup. This backup
   will be used to automatically restore any additional replicas that you run,
   before they connect themselves to the master and catch up on replication.
   If an existing tablet goes down and comes back up without its data, it will
   also automatically restore from the latest backup and then resume replication.

   ```
   vitess/examples/local$ ./lvtctl.sh Backup test-0000000102
   ```

   After the backup completes, you can list available backups for the shard:

   ```
   vitess/examples/local$ ./lvtctl.sh ListBackups test_keyspace/0
   ### example output:
   # 2016-05-06.072724.test-0000000102
   ```

   **Note:** In this single-server example setup, backups are stored at
   `$VTDATAROOT/backups`. In a multi-server deployment, you would usually mount
   an NFS directory there. You can also change the location by setting the
   `-file_backup_storage_root` flag on *vtctld* and *vttablet*, as demonstrated
   in `vtctld-up.sh` and `vttablet-up.sh`.
9. **Initialize Vitess Routing Schema**

   In the examples, we are just using a single database with no specific
   configuration. So we just need to make that (empty) configuration visible
   for serving. This is done by running the following command:

   ```
   vitess/examples/local$ ./lvtctl.sh RebuildVSchemaGraph
   ```

   (As it works, this command will not display any output.)
10. **Start vtgate**

    Vitess uses *vtgate* to route each client query to
    the correct *vttablet*. This local example runs a
    single *vtgate* instance, though a real deployment
    would likely run multiple *vtgate* instances to share
    the load.

    ```
    vitess/examples/local$ ./vtgate-up.sh
    ```

### Run a Client Application

The `client.py` file is a simple sample application
that connects to *vtgate* and executes some queries.
To run it, you need to either:

- Add the Vitess Python packages to your `PYTHONPATH`.

  or
- Use the `client.sh` wrapper script, which temporarily
  sets up the environment and then runs `client.py`.

  ```
  vitess/examples/local$ ./client.sh
  ### example output:
  # Inserting into master...
  # Reading from master...
  # (5L, 1462510331910124032L, 'V is for speed')
  # (15L, 1462519383758071808L, 'V is for speed')
  # (42L, 1462510369213753088L, 'V is for speed')
  # ...
  ```

There are also sample clients in the same directory for Java, PHP, and Go.
See the comments at the top of each sample file for usage instructions.

### Try Vitess resharding

Now that you have a full Vitess stack running, you may want to go on to the
Horizontal Sharding workflow guide
or Horizontal Sharding codelab
(if you prefer to run each step manually through commands) to try out
dynamic resharding.

If so, you can skip the tear-down since the sharding guide picks up right here.
If not, continue to the clean-up steps below.

### Tear down the cluster

Each `-up.sh` script has a corresponding `-down.sh` script to stop the servers.

```
vitess/examples/local$ ./vtgate-down.sh
vitess/examples/local$ ./vttablet-down.sh
vitess/examples/local$ ./vtctld-down.sh
vitess/examples/local$ ./zk-down.sh
```

Note that the `-down.sh` scripts will leave behind any data files created.
If you're done with this example data, you can clear out the contents of `VTDATAROOT`:

```
$ cd $VTDATAROOT
/path/to/vtdataroot$ rm -rf *
```

## Troubleshooting

If anything goes wrong, check the logs in your `$VTDATAROOT/tmp` directory
for error messages. There are also some tablet-specific logs, as well as
MySQL logs in the various `$VTDATAROOT/vt_*` directories.

If you need help diagnosing a problem, send a message to our
mailing list.
In addition to any errors you see at the command-line, it would also help to
upload an archive of your `VTDATAROOT` directory to a file sharing service
and provide a link to it.

Contact: vitess@googlegroups.com  **·**  
Announcements  **·**  
© 2017 Vitess powered by Google Inc
